# The clinicopathologic observation, c-KIT gene mutation and clonal status of gastrointestinal stromal tumor in the sacrum

**DOI:** 10.1186/1471-230X-9-43

**Published:** 2009-06-06

**Authors:** Li Gong, Yan-Hong Li, Hua-Dong Zhao, Jian-Ye Zhao, Wei Zhang

**Affiliations:** 1 Department of Pathology, Tangdu Hospital, the Fourth Military Medical University, Xi'an 710038, Shaanxi Province, PR China; 2 Department of Gynaecology and Obstetrics, Tangdu Hospital, the Fourth Military Medical University, Xi'an 710038, Shaanxi Province, PR China; 3 Department of General Surgery, Tangdu Hospital, the Fourth Military Medical University, Xi'an 710038, Shaanxi Province, PR China

## Abstract

**Background:**

It is very rare that gastrointestinal stromal tumor (GIST) occurs in the sacrum. Only one case of GIST occuring in the sacral region, with intracranial metastasis, has been reported in the literature. Moreover, only few cases have been published in literature about its clonal origin.

**Case presentation:**

In this report, we present a rare case of GIST occuring in the sacrum and describe its clinicopathologic features, c-KIT gene mutation and clonal status. Microscopically, the lesion was composed of spindle cells arranged in cords, knitted and whirlpool patterns. Trabecula of bone were found in the lesion. The cytoplasm of tumor cells were abundant, and the nuclei were fusiform. Mitotic figures were rare. Immunohistochemically, the tumor cells showed positive reactivity for CD117 and CD34. On mutation analysis, a c-KIT gene mutation was found in exon 11. The result of clonal analysis demonstrated that the GIST was monoclonal.

**Conclusion:**

In summary, we showed that tumor material, phenotypically identical with GISTs was found in the sacrum. It is difficult to differentiate GISTs from other spindle cell tumors, hence the need for immunohistochemistry, the examination of c-KIT gene amplification and sequencing.

## Background

Gastrointestinal stromal tumors (GISTs) are the most common mesenchymal tumors of the gastrointestinal tract. They are supposed to arise from the Cajal's cells expressing c-KIT [1]. This type of tumor is considered to be a gastrointestinal tract primary, non-epithelial, non-lymphatic, non-smooth muscle and non-schwannoma neoplasm. Its most common anatomic sites of origin are the stomach (60–70%), small intestine (20–30%), colon and rectum (5%), abdominal cavity, peritoneum and omentum (5%), esophagus (<5%) and the retroperitoneal space (<3%) [2-4]. However, it is very rare that GISTs occur in sacrum. Only one case of GIST of the sacral region with intracranial metastasis has been reported in the literature [5].

In this report we present a rare case of GIST occuring in the sacrum, describing and discussing its histopathological characteristic and c-KIT gene mutation as an aid for the pathologist. We suggest that GIST should be considered when a spindle cells tumor with atypical immunological phenotype occurs in the bone. In addition, the examination of c-KIT gene amplification and sequencing should be carried out to further confirm its diagnosis.

## Case presentation and Methods

### Case presentation

**Figure 1:**
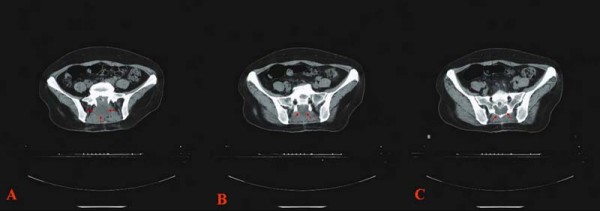
**Computed tomography (CT) scan of the abdomen and plevis demonstrated a small (2 × 1.5 × 0.8 cm) low-density, well circumscribed mass in the sacrum, without evidence of tumor infitration of adjacent structures (three different CT-slice, A, B, and C)**.

A 50-year-old female patient with the history of lower left limb pain and dyschesia for three months was admitted to the department of Bone, Tangdu Hospital, the Fourth Military Medical University, Xi'an, ShaanXi Province, China. There was no history of swelling, anesthesia, limitation of activity, hemafecia or crissum pain. A computed tomography (CT) scan of the abdoman and pelvis demonstrated a small (2 × 1.5 × 0.8 cm) low-density, well circumscribed mass in the sacrum, without evidence of tumor infiltration of adjacent structures(Figure. 1). Then, a resection was carried out. Written informed consent was obtained from the patient and the protocol was approved by the Institutional Ethics Committee of the Fourth Military Medical University based on the Helsinki Declaration.

## Methods

### Immunohistochemistry

Immunohistochemical staining was carried out using a streptavidin-labeled peroxidase (S-P) kit (KIT9730) according to the manufacturer's instructions. The primary antibodies used in this study included those against CD34, Desmin, neuronal specific enolase (NSE), nerve fiber (NF), placental alkaline phosphatase (PLAP) and glial fibrillary acidic protein (GFAP) for mouse anti-human monoclonal antibodies (mAb), and CD117, S-100 protein, smooth muscle actin (SM-actin), SC-actin, and to exclude melanoma HMB45 for rabbit anti-human polyclonal antibodies, as well as vimentin for mouse anti-pig mAb. All of the reagents for immunostaining were supplied by Maxim Biotechnology Corporation Limited, Fuzhou, China.

### Microdissection and DNA extraction

Eight 10 μm sections from formalin-fixed, paraffin embedded tissue samples were used for DNA extraction. After H/E staining, the sections were covered with 100% glycerol. Lesions in the sections were dissected using a syringe needle under an inverted microscope after observing their histological appearance. Tissue samples were collected in 100% ethanol in 1.5-mL tubes. For each dissected neoplasm, the surrounding fibrous connective tissue (with the same surface area) was also isolated and analyzed as a control. Collected samples were dehydrated three times with 100% ethanol and then dried at room temperature. Genomic DNA was extracted using a QIAamp Kit (Qiagen, Mannheim, Germany) according to the manufacturer's instructions.

### c-KIT mutation analysis

Exons 9, 11, 13 and 17 of the c-KIT gene were evaluated for the presence of mutations by PCR amplification and direct sequencing. The primer pairs used for PCR amplification and direct sequencing are given in Table 1. Briefly, the PCR reaction was carried out in a final volume of 50 μl, under the following conditions: 4 μL of 10 mM dNTP (Gibco BRL, Life Technologies, Inc., Gaithersburg, MD, USA), forward and reverse primers (0.4 pmol each), 5 μL of 10× buffer, 1.5 μL of 50 mM MgCl_2 _and 2.5 U of Taq DNA polymerase (Gibco BRL). The amplification was conducted using a PT-200 thermocycler (MJ Research, Inc., Watertown, MA, USA) for 35 cycles (95°C for 30 sec, 56°C or 60°C for 40 sec, and 72°C for 30 sec). Amplification products were separated by 2% agarose ethidium bromide gel electrophoresis to confirm correct amplification. Then, direct sequencing was performed; the results showed no mutation in exon 9, 13 and 17 expect for exon 11. Thus, the PCR products for exon 11 was ligated to the pGEM-T plasmid vector, the recombinant vector was transformed into E. coli DH5alpha, and the positive clones were selected by blue/white screening. Restriction enzyme digestion with EcoR I and BamH I, gel electrophoresis analysis, followed by sequencing of the digested product were performed for identification of the recombinant product. The informative plasmid was then sent to Shanghai Biotechnology Co. Ltd., for purification and sequencing.

**Table 1:** c-KIT gene oligonucleotide primer

c-KIT exon sequence (5'-3')	length of PCR product (bp)	annealing temperature (°C)
**9 (F) **5'-TCC TAG AGT AAG CCA GGG CTT T-3'	283	56
**9 (R) **5'-TGG TAG ACA GAG CCT AAA CAT CC-3'		
**11 (F) **5'-CTG AGA CAA TAA TTA TTA AAA GGT GA-3'	227	60
**11 (R) **5'-TTA TGT GTA CCC AAA AAG GTG ACA-3'		
**13 (F) **5'-GCT TGA CAT CAG TTT GCC AG-3'	193	60
**13 (R) **5'-AAA GGC AGC TTG GAC ACG GCT TTA-3'		
**17 (F) **5'-TAC AAG TTA AAA TGA ATT TAA ATG GT-3'	228	56
**17 (R) **5'-AAG TTG AAA CTA AAA ATC CTT TGC-3'		

### PCR amplification for clonal assay

Nested PCR was used for amplification and detection of single nucleotide polymorphism (SNP) sites in the phosphoglycerate kinase (PGK) and the length polymorphism of CAG short-tandem repeat (STR) in exon 1 of the androgen receptor (AR) gene loci, two pairs of primers to amplify the PGK gene were used: PGK1A, 5'-CTG TTC CTG CCC GCG CGG TGT TCC GCA TTC-3'; PGK1B, 5'-ACG CCT GTT ACG TAA GCT CTG CAG GCC TCC-3'; PGK2A, 5'-AGC TGG ACG TTA AAG GGA AGC GGG TCG TTA-3'; PGK2B, 5'-TAC TCC TGA AGT TAA ATC AAC ATC CTC TTG-3'. The genomic DNA extracts from lesions and surrounding tissue, 10 μL each, were incubated with 5 U of *Hpa *II (Promega, Madison, WI, USA) at 37°C for 3 h in a volume of 20 μL containing 0.2 μL of 10 g/L bovine serum albumin (BSA) and 2 μL of 10× reaction buffer. The digested DNA samples, 5 μL each, were then subjected to nested PCR. The reaction mixture was 50 μL in volume, containing 4 μL of 10 mM dNTP (Gibco BRL, Life Technologies, Inc., Gaithersburg, MD, USA), primers PGK1A and PGK1B (0.4 pmol each), 5 μL of 10× buffer, 1.5 μL of 50 mM MgCl_2 _and 2.5U of Taq DNA polymerase (Gibco BRL). The amplification was conducted using a PT-200 thermocycler (MJ Research, Inc., Watertown, MA, USA) for 35 cycles (94°C for 40 sec, 58°C for 50 sec, and 72°C for 1 min). The first round of PCR products (5 μL) were diluted (1:20) and used as templates for a second PCR reaction using the primers PGK2A and PGK2B. The amplification procedure was the same as for the first round, except that the annealing temperature was 56°C. The PCR products were digested with 5 U Bst XI at 48°C for 8–10 h in a 20 μL mixture containing 0.2 μL of 10 g/L BSA and 2 μL of 10× reaction buffer. The digested products were resolved in 2% (g/mL) agarose gel containing ethidium bromide (0.2 mg/L).

For the AR gene, two pairs of primers and amplication condition were used according to the previous described [6]. The amplification efficiency was checked by resolving PCR reaction aliquots on 2% agarose gels. The PCR products, 4 μL for each, were also mixed with the same volume of loading buffer (1 g/L xylene cyanole, 1 g/L bromophenol blue, in formamide), and resolved on an 8% polyacrylamide gel containing 8 mol/L urea using the Mini-VE system (Amersham Biosciences, San Francisco, CA, USA) at 120 V for 4 h. Bands were visualized using silver staining.

### Analysis and assessment of PCR products

Images of PCR gels were recorded and the intensities of the PCR bands for both alleles were quantitated using an image-analyzing system (LabWorks 3.0, UVP, Cambridge, UK). A reduction in fluorescence intensity of at least 50% for either band, as compared to the intensity of the bands obtained in the absence of *Hpa *II or *Hha *I digestion, were used as indicators of a loss of X chromosome inactivation mosaicism [7]. A corrected ratio (CR) was calculated by dividing the ratio of the upper-band intensity to the lower-band intensity, or vice versa, of the same sample before and after digestion to give a CR value >1. In the present study, a CR value ≥ 2 was used to define a loss of X chromosome inactivation mosaicism.

## Results

### Pathological observation

**Figure 2:**
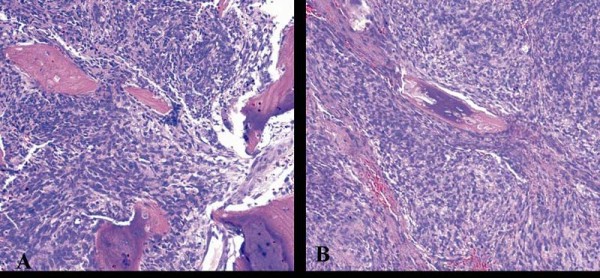
**Microscopically, the lesion was composed of spindle cells arranged in cords, knit and whirlpool patterns**. Trabecula of bone was found in them (2A, 2B Original magnification 200×).

**Figure 3:**
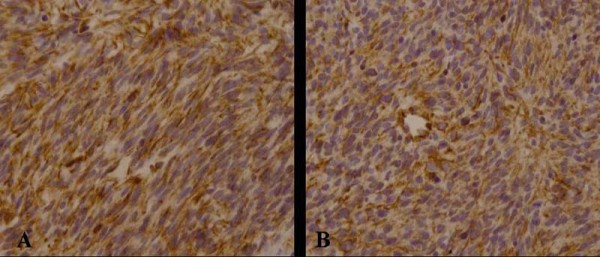
**The tumor cells were positive for CD117 (3A, Original magnification 200×) and CD34 (3B, Original magnification 200×)**.

The resected irregular tissue was 4.0 cm × 2.5 cm × 0.5 cm in size, the cut surface being grayish-white and the texture being moderate. Microscopically, the lesion was composed of spindle cells arranged in cords, knitted and whirlpool patterns. Trabecula of bone were found in the lesion (Figure 2A, 2B). The cytoplasm of tumor cells were abundant, and the nuclei were fusiform. Evidence of mitosis was not readily apparent. The tumor tissues showed a positive reaction for CD117 (Figure 3A), CD34 (Figure 3B) and vimentin, but not for desmin, PLAP, S-100, SM-actin, NF, GFAP, NSE, HMB-45, and SC-actin.

### The amplification and detection of mutation of exons 9, 11, 13, 17 of the c-KIT gene

**Figure 4:**
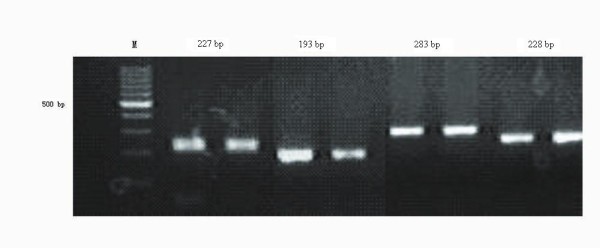
**The results of 2% agarose gels showed that there was a band at 283 bp, 227 bp, 193 bp and 228 bp, respectively**.

**Figure 5:**
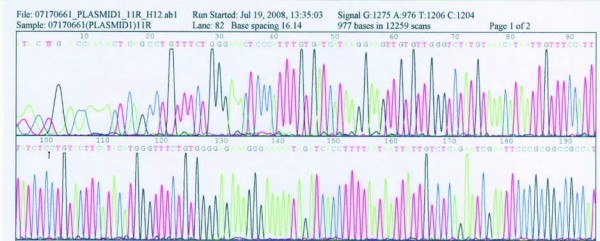
**The result of DNA sequencing demonstrated that the c-kit gene mutation was only found in exon 11, namely the deletion of 15 bp (CTC AAC AAC CTT CCA) was found at position 100 bp**.

The amplification efficiency for exon 9, 11, 13 and 17 of the c-KIT gene were checked by resolving PCR reaction aliquots on 2% agarose gels. Bands were observed at 283 bp, 227 bp, 193 bp and 228 bp, respectively (Figure 4), which further confirmed the diagnosis of GIST. Moreover, mutations of exons 9, 11, 13 and 17 of the c-KIT gene were examined according to the PCR methods. The result demonstrated that the c-KIT gene mutation was only found in exon 11, namely the deletion of 15 bp (CTC AAC AAC CTT CCA) was found at position 100(Figure 5), but not in the following domains (exon 9, 13 and 17).

### Clonality determination

**Figure 6:**
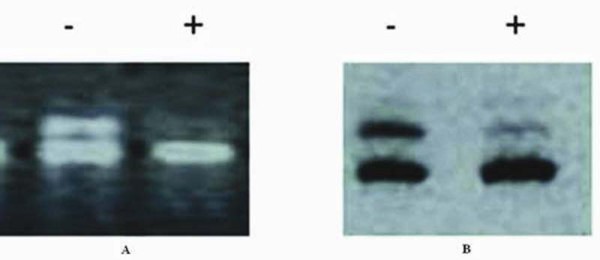
**When the DNA samples of the lesion tissue were digested with *Hpa *II or *Hha* I**. a) the upper bands exhibited an obviously reduced intensity or disappeared for PGK gene loci. -, pretreated without *Hpa *II; +, pretreated with *Hpa *II; M, DNA marker. b) the upper bands exhibited an obviously reduced intensity or disappeared for AR gene loci. -, pretreated without *Hha *I; +, pretreated with *Hha *I; M, DNA marker.

The clonality assay demonstrated their polymorphism for the samples at PGK and AR loci. On the PGK and AR PCR gel pictures, DNA samples of the tissue analysed without *Hpa *II or *Hha *I digestion showed two bands of equal intensity. when DNA samples of the lesions were digested with *Hpa *II or *Hha *I, the upper bands exhibited an obviously reduced intensity or had disappeared (Figure 6A, 6B). Monoclonality was demonstrated for the lesions, suggesting that they were neoplastic lesions. However, the intensities of the two bands were equal for the surrounding fibrous connective tissue treated with or without *Hpa *II or *Hha *I. The results indicate that GIST is a neoplastic lesion.

## Discussion and Conclusion

Gastrointestinal stromal tumors (GISTs) are the most common mesenchymal tumors of the gastrointestinal tract with an incidence of 10–20 cases per million of which almost one third are deemed malignant [8]. The usual age at presentation is around 60 years, and this tumor is predominantly seen in Caucasians with little gender difference. GIST may present anywhere in the gastrointestinal tract, omentum, or mesentery. The most common sites are the stomach (60%), small intestine (15%), colon and rectum (5%), other abdominal organs including mesentery and omentum (5%) [9]. However, it is very rare that GIST occurs in the sacrum.

The diagnosis of GISTs mostly relies on histopathological features and the immunohistochemical phenotype, and the most important immunohistochemical feature is the positivity of the tumor cells for CD117 and CD34. It has been reported in the literature that 90–95% of GISTs express CD117, 60–70% express CD34, 30–40% SM-actin and only 5% are positive for S-100 protein [10]. GIST could be differentiated from other mesenchymal tumors, such as schwannoma or leiomyoma, according to its distinct immunohistochemical characteristics. Based on histopathological observation and immunohistochemical staining in the current case, the tumor cells were found positive for CD117 and CD34 and showed typical morphological characteristics. Moreover, the results of PCR amplification and detection of mutation of the c-KIT gene showed that the c-KIT gene mutation occurred in exon 11, but not in other domains investigated (exon 9, 13 and 17), in support of a diagnosis of GIST in this case. However, it must be differentiated from germ cells tumors from female, such as dysgerminoma of the ovary and mixed germ cell tumors. Because their tumor cells are not only positive for CD117 [11], but also there has been reported a frequent presence of KIT mutations in them [12]. But it is different that a typical dysgerminoma component has uniform individual tumor cells. These show the usual microscopic findings, which are round nuclei with granular chromatin, distinct nucleoli, clear to finely granular cytoplasm containing abundant glycogen and distinct cell borders. In addition, lymphocytes (most of which are of T-cell type) may be found in stromal fibrous strands. Immunohistochemically, the tumor cells are positive for PLAP and CD117, but negative for CD34. The above of characteristics are different from that of GIST. Thus, the diagnosis of GIST is reliable.

However, the results of our analysis did not identify the tumor as being primary or secondary. When a GIST occurs in bone, it should immediately be suspected as being a metastatic or invasive lesion from the gastrointestinal tract. In the current study, after the diagnosis of GIST was made, a series of examinations, including computerized tomographic (CT) scanning and magnetic resonance imaging (MRI) on liver, cholecyst, pancreas, spleen, kidney and ovary, and especially an electronic endoscopy on digestive tract, were performed on the patient, but no lesion was found. The question then becomes whether the original lesion had disappeared or this GIST had occurred primarily in the bone. The possibility of primary origin was worth investigating further.

Monoclonality is one of the main features of most tumors, whereas normal and reactive hyperplastic lesions are polyclonal [13]. The clonality assay is based on X-chromosome inactivation mosaicism and polymorphism at the PGK and AR loci in female somatic cells. There are two X chromosomes within each female somatic cell, one of which is inactivated randomly by methylation during early embryogenesis, while the other preserves its genetic activity throughout life [14]. PGK gene polymorphism showed there was a single nucleotide polymorphic site identified by *Bst *XI located downstream of the methylation site [15]. AR polymorphism shows different lengths of the CAG short-tandem repeat (STR) at exon 1 [16]. After digestion with the methylation-sensitive restriction enzymes *Hpa *II or *Hha *I, the normal and reactive hyperplastic tissues with polymorphism showed two alleles with equal intensity. Neoplastic tissues showed only one, or one of two alleles with obviously reduced intensity [17].

GIST has been considered as a mesenchymal tumor in recent years. Only few studies have been reported in the literature about its clonal origin [18,19]. In our analysis of the clonal status of a case of multiple GISTs occurring in the peritoneum, GIST appeared to be monoclonal, and 15 different tumor nodules showed loss of the identical X chromosomal inactivation mosaicism. This indicated a common clonal origin for the tumors [20]. The results of the clonal assay for the current case were similar to those previously described, thus, confirming further the neoplastic nature of the GIST examined in this study.

## Abbreviations

AR: androgen receptor; GIST: gastrointestinal stromal tumor; CT: computed tomography; SNP: single nucleotide polymorphism; PGK: phosphoglycerate kinase; STR: short-tandem repeat; CR: corrected ratio.

## Competing interests

The authors declare that they have no competing interests.

## Authors' contributions

GL carried out the whole study and drafted the manuscript. LYH participated in the design of the study. ZHD participated in drafting the manuscript. ZJY carried out pathological observation. ZW participated in its design and coordination and helped to draft the manuscript. All authors read and approved the final manuscript.

## Pre-publication history

The pre-publication history for this paper can be accessed here:

http://www.biomedcentral.com/1471-230X/9/43/prepub
